# Axonal transport deficit in a *KIF5A*^*–/–*^ mouse model

**DOI:** 10.1007/s10048-012-0324-y

**Published:** 2012-04-01

**Authors:** Kathrin N. Karle, Diana Möckel, Evan Reid, Ludger Schöls

**Affiliations:** 1 Department of Neurology, Hertie Institute for Clinical Brain Research and German Center of Neurodegenerative Diseases, University of Tübingen, Hoppe-Seyler-Str. 3, 72076 Tübingen, Germany; 2 Department of Medical Genetics, Cambridge Institute for Medical Research, Addenbrooke’s Hospital, University of Cambridge, Wellcome Trust/MRC Building, Cambridge, CB2 OXY UK

**Keywords:** Motor neuron, KIF5A, Axonal transport, Axonal outgrowth, Mitochondria, Hereditary spastic paraplegia (HSP)

## Abstract

Hereditary spastic paraplegia (HSP) is a neurodegenerative disorder preferentially affecting the longest corticospinal axons. More than 40 HSP genetic loci have been identified, among them SPG10, an autosomal dominant HSP caused by point mutations in the neuronal kinesin heavy chain protein KIF5A. Constitutive KIF5A knockout (*KIF5A* ^*–/–*^) mice die early after birth. In these mice, lungs were unexpanded, and cell bodies of lower motor neurons in the spinal cord swollen, but the pathomechanism remained unclear. To gain insights into the pathophysiology, we characterized survival, outgrowth, and function in primary motor and sensory neuron cultures from *KIF5A* ^*–/–*^ mice. Absence of KIF5A reduced survival in motor neurons, but not in sensory neurons. Outgrowth of axons and dendrites was remarkably diminished in *KIF5A* ^*–/–*^ motor neurons. The number of axonal branches was reduced, whereas the number of dendrites was not altered. In *KIF5A* ^*–/–*^ sensory neurons, neurite outgrowth was decreased but the number of neurites remained unchanged. In motor neurons maximum and average velocity of mitochondrial transport was reduced both in anterograde and retrograde direction. Our results point out a role of KIF5A in process outgrowth and axonal transport of mitochondria, affecting motor neurons more severely than sensory neurons. This gives pathophysiological insights into KIF5A associated HSP, and matches the clinical findings of predominant degeneration of the longest axons of the corticospinal tract.

**Electronic supplementary material:**

The online version of this article (doi:10.1007/s10048-012-0324-y) contains supplementary material, which is available to authorized users.

## Introduction

The hereditary spastic paraplegias (HSPs) comprise a growing number of clinically and genetically heterogeneous diseases. Their common feature is axonal degeneration of the corticospinal tract in a length-dependent manner, so that the longest axons innervating lower motor neurons that supply the leg muscles are affected, whereas the shorter fibers that innervate lower motor neurons supplying the arms are mainly spared. Clinically, this leads to progressive gait disturbance due to lower limb spasticity [1–3]. More than 40 HSP loci and 20 HSP genes are registered in the HUGO Gene Nomenclature Committee database [4]. Autosomal dominant, autosomal recessive, and X-linked modes of inheritance occur in HSP [5]. Increasing knowledge about physiological functions of genes mutated in HSP has highlighted a few common pathogenic mechanisms, such as myelin sheath formation, mitochondrial function, and membrane trafficking and transport [6–8]. Axons rely on many of these functions in a length-dependent manner, offering an explanation why motor neurons to the legs with an axon length up to 1 m are especially sensitive to dysfunction.

SPG10 is an autosomal dominantly inherited subtype of HSP that is caused by mutations in the neuronal motor protein kinesin heavy chain (KHC) KIF5A. SPG10 accounts for 3 to 5 % of autosomal dominant HSPs in European families [9, 10]. As in most HSP subtypes, disease onset varies from early childhood to adulthood, and the disease may cause pure spastic paraplegia, or alternatively forms complicated by cognitive dysfunction, peripheral neuropathy, parkinsonism, and/or epilepsy. Nearly all mutations causing SPG10 are heterozygous missense mutations in the motor domain of KIF5A [9–14].

The kinesin superfamily provides motors for ATP-dependent transport along microtubules, generally in the plus end direction (which in axons is the anterograde direction). Kinesin-1, formerly called conventional kinesin, acts as a motor for fast axonal transport of membranous organelles (50–200 mm per day) as well as for slow axonal transport of cytoplasmic proteins (0.1–3 mm per day) [15–17]. Kinesin-1 is a heterotetramer consisting of two kinesin heavy chains (KIF5A, KIF5B, or KIF5C), and two kinesin light chains [18]. Kinesin heavy chains as well as kinesin light chains only homodimerize, whereas there is no specificity in the interaction between heavy and light chains [19]. The heavy chains contain the motor domain, cargo specificity is thought to be mediated mainly via kinesin light chains and/or additional linker proteins, e.g., milton and miro are likely to act as linker proteins binding mitochondria to kinesin heavy chain protein [20]. Other cargoes transported by kinesin-1 include lysosomes [21], membrane-associated SNARE proteins [22], syntaxin-1-containing vesicles [23], tubulin [24], apolipoprotein E receptor 2 [25], and phosphorylated amyloid precursor protein (APP) [26]. Circumstantial evidence that KIF5A is involved in mitochondrial transport comes from primary extraembryonic membrane cells lacking the *KIF5B* gene. The abnormal perinuclear clustering of mitochondria in these cells can be rescued by overexpression of the *KIF5A* gene [27]. In vitro experiments with KIF5A proteins carrying human SPG10 missense mutations of the motor domain revealed reduced transport velocity and reduced binding on microtubules respectively [28].

*KIF5A knockout* (*KO*) mice die shortly after birth. The amount of KIF5B and KIF5C was not significantly changed. Histologically, lungs were not well expanded, and cell bodies of lower motor neurons in the spinal cord swollen whereas the brain did not seem to be affected. To overcome perinatal lethality conditional knockout mice were generated with neuronal knockout of KIF5A induced by synapsin-promoted Cre-recombinase transgene. These mice showed seizures, hind limb paralysis, and sensory neuron degeneration in later stages. Histological analysis of cell bodies from sensory neurons revealed an accumulation of neurofilament (L, M, H), in Western Blots of DRGs the amount of neurofilament protein was increased, whereas the amount of markers for fast axonal transport (APP, Rab3, synaptophysin) was not changed [29]. In superior cervical ganglia neurons, frequency and velocity of neurofilament movements were reduced both in anterograde and retrograde direction [30]. In a mouse model with the human N256S mutation in the *KIF5A* gene cortical neurons revealed an increased retrograde velocity of neurofilament M transport, whereas anterograde velocity was not affected. The frequency of anterograde and retrograde movements was decreased [31]. But probably neurofilament is not the only important cargo transported by KIF5A.

In this work, we established primary cell cultures of motor and sensory neurons of constitutive *KIF5A KO* mice and characterized vitality, morphology, and function. By live cell imaging, we revealed an axonal transport defect of mitochondria. These results can improve insights into mechanisms underlying the length-dependent axonal degeneration and selective damage of motor neurons in SPG10.

## Materials and methods

### Histology of mouse brain, spinal cord, and muscle

*KIF5A* ^*+/–*^ mice were obtained from Mutant Mouse Regional Resource Centers, University of California, Davis, USA. Wildtype C57Bl/6N mice were purchased from Charles River Laboratories (Sulzfeld, Germany). For histological analysis embryos were dissected from pregnant mice at embryonic day (E) 18.5. Brain, spinal cord, and proximal leg muscle were isolated and fixed in 4 % paraformaldehyde for 24 hours. After dehydration tissues were embedded in paraffin. 5 μm sections of brain and spinal cord were stained with cresyl violet (MEDITE® GmbH, Burgdorf, Germany) whereas 5 μm muscle sections were treated with hematoxylin eosin (MEDITE® GmbH). Images were acquired with a CCD camera (Axio Imager Z1, AxioCam MRc, Carl Zeiss MicroImaging GmbH, Jena, Germany).

For morphological assessment of spinal motor neurons the size of cell bodies and nuclei were analyzed in at least 36 cells from three independent experiments. Statistical evaluation was performed by ANOVA followed by Bonferroni correction (SPSS 17.0, SPSS Inc., Chicago, USA).

### Mouse embryonic motor and sensory neuron culture

Embryos were isolated from pregnant mice at E 12.5. The lumbar spinal cord and the laterally adjacent DRGs were dissected and transferred to HBSS (invitrogen™, Carlsbad, USA) and 1× PBS (invitrogen™) respectively. The trypsin reaction (0.05 %, Biochrom AG, Berlin, Germany) was stopped in spinal cord tissue after 15 min with 0.01 % trypsin inhibitor (Sigma-Aldrich Co., St. Louis, MO, USA), and in DRGs after 35 min with HAMS F14 Powder Medium (invitrogen™), enriched with 10 % heat-inactivated horse serum (Linaris Biologische Produkte GmbH, Wertheim-Bettingen, Germany) and 35 mM KCl. Tissues were triturated.

Motor neurons of the spinal cord were isolated via Lectin antibody (20 μg/ml, Sigma-Aldrich) that has been attached to 24 well CELLSTAR® plates (Greiner Bio-One GmbH, Frickenhausen, Germany). After panning for 30 min, the supernatant was removed, and adherent motor neurons washed three times with Neurobasal medium (invitrogen™). Afterwards motor neurons were resolved with 30 mM KCl and 137 mM NaCl (in aqua dest.). After centrifugation (400 g, 5 min, Multifuge3 S-R, Heraeus Holding GmbH, Hanau, Germany), and removal of the supernatant, motor neurons were resuspended in Neurobasal medium with 10 % heat-inactivated horse serum, 2 % B27 supplement (invitrogen™) and 500 μM GlutaMAX-I-Supplement (invitrogen™). CNTF (upstate®, Millipore™, Billerica, USA) and BDNF (CHEMICON®, Millipore™) were added in a final concentration of 1 ng/ml. Cells were plated on six-well cell culture CELLSTAR® plates (Greiner Bio-One GmbH) with glass coverslips (22 mm diameter, Carl Roth GmbH + Co. KG, Karlsruhe, Germany) and on Lab-Tek™ 8 Chamber Slide™ system (Nalge Nunc International, Rochester, USA) precoated with 1× poly-d-lysine (Sigma-Aldrich) and natural mouse laminin (Sigma-Aldrich, 2,5 μg/ml) at a density of 2,000 cells/cm^2^. Cells were incubated at 37°C and 5 % CO_2_. Fifty percent of the medium was replaced every day.

To suppress growth of non-neuronal cells, sensory neurons were pre-plated after trituration on 24-well plates in HAMS F14 Powder Medium with 10 % heat-inactivated horse serum and 35 mM KCl. After 2 h the supernatant was transferred to six-well plates precoated with poly-l-ornithine and laminin. BDNF, GDNF (Alomone Labs Ltd., Jerusalem, Israel), NGF 2.5S (Alomone Labs Ltd.), and NT-3 (Alomone Labs Ltd.) were added in a final concentration of 1 ng/ml. Cells were incubated at 37°C and 5 % CO_2_.

### Genotyping of *KIF5A KO* mice

Genomic DNA from mouse tissues was isolated by standard methods. The following primers were used to detect the *KIF5A* gene: KIF5AI_P1 5′ GAT ACT CCA AGG CTG GGA ACA TA 3′, KIF5AI_P2 5′ TGT GGA GGT CAG AGG TCA AGT 3′, loxP5AI_P3 5′ CGG TAC CCG GGG ATC AAT TCG AG 3′ (metabion international AG, Martinsried, Germany). PCR mix includes 1 μl of DNA (concentration 100–200 ng/μl), 2 μl 10× buffer, 0.4 μl dNTPs (10 mM), 0.1 μl SAWADY hot taq-DNA-polymerase (5 U/μl, PEQLAB Biotechnologie GmbH, Erlangen, Germany), 4 μl Enhancer (PEQLAB Biotechnologie GmbH), 0.2 μl KIF5AI_P1 (10 pmol/μl), 0.1 μl KIF5A_P2 (10 pmol/μl), 0.1 μl loxP5AI_P3 (10 pmol/μl), water dest. added to 20 μl. PCR conditions (Bio-Rad Dyad Thermal Cycler, Bio-Rad Laboratories Inc., Hercules, USA) were activation of taq-DNA-polymerase at 95°C for 15 min, followed by 35 cycles with denaturation at 94°C for 45 s, annealing at 60°C for 45 s, elongation at 72°C for 1 min, and a last elongation at 72°C for 10 min PCR products are a wildtype band (P1/P2) at ∼450 bp and a mutated band (P1/P3) at ∼300 bp.

### Determination of motor and sensory neuron survival

The first counting was done 4 h after plating the cells, when they were attached to the bottom. Then, every day the number of surviving cells in ten fields of view (1.16 mm^2^) was counted under a phase-contrast microscope (Olympus Deutschland GmbH, Hamburg, Germany). The number of initially counted cells was set 100 % (day 0). The percentage of surviving cells was calculated for every day in culture. The results from at least four independent experiments were pooled. So for each genotype at least 11 individuals were counted, and are given as mean and standard error of mean. Statistical significance of differences was assessed by ANOVA followed by Bonferroni correction (SPSS Statistics 17.0, SPSS Inc.).

### Immunocytochemistry of motor and sensory neurons

After a definite number of days in culture motor and sensory neurons on glass coverslips were fixed with 4 % paraformaldehyde (Carl Roth GmbH + Co. KG) for 30 min and washed three times with 1× PBS. After treatment with 0.1 % Igepal CA-630 (Sigma-Aldrich) for 30 min unspecific bindings were blocked with 10 % bovine serum albumin (BSA, Carl Roth GmbH + Co. KG, solved in 1× TBS-T) for 60 min Cells were incubated for 60 min with the following primary antibodies dissolved in 1 % BSA in 1× TBS-T: islet-1 (anti-rabbit, polyclonal, 1:500, Biozol Diagnostika Vertrieb GmbH, Eching, Germany), phospho-tau (p-tau, anti-rabbit, polyclonal, 1:1,000, Biozol Diagnostika Vertrieb GmbH), microtubule-associated protein 2ab (MAP2ab, anti-mouse, monoclonal, 1:500, Acris Antibodies GmbH, Hiddenhausen, Germany), acetylcholine transferase (AChT, anti-rabbit, polyclonal, 1:500, Chemicon®, Millipore™), alpha-tubulin (anti-mouse, monoclonal, 1:1,000, Sigma-Aldrich), neurofilament L (anti-rabbit, polyclonal, 1:500, Millipore™). The primary antibody was removed by washing the coverslips three times with 1× TBS-T. Secondary antibodies (dissolved in 1 % BSA in 1× TBS-T) were added for 45 min in the dark: Alexa Fluor 488 goat anti-rabbit and anti-mouse IgG, Alexa Fluor 568 anti-rabbit and anti-mouse IgG (1:1,000, Molecular Probes®, invitrogen™). Nuclei were stained with Hoechst 33258 (1:5,000, Sigma-Aldrich).

After washing the coverslips three times with 1× TBS-T, they were embedded in fluorescent mounting medium (Dako Denmark A/S, Glostrup, Denmark), and stored at 4°C in the dark. Immunofluorescence was visualized with a fluorescence microscope (Axio Imager Z1, AxioCam MRm, Carl Zeiss MicroImaging GmbH, Jena, Germany). All images were obtained using identical camera, microscope, and imaging criteria such as gain, brightness and contrast, and exposure time. Morphological assessment of axonal swellings was performed in three independent experiments, for each genotype at least 145 cells were analyzed. The results are given as mean and standard deviation. For outgrowth analysis, axons of motor neurons were identified via positive anti-phospho-tau staining, dendrites via positive anti-MAP2ab staining. The following outgrowth parameters were measured with the Neurolucida 8 software (MBF Bioscience, Williston, USA), and are given as mean and standard deviation: longest axonal branch, total axon length (including all branches), longest dendrite, total dendrite length (of one cell), number of axonal branches, number of dendrites, and cell body area. An example for the outgrowth analysis is shown in Online Resource 1. In sensory neurons, anti-phospho-tau positive neurite length (total neurite length, longest neurite) was measured. Results from three independent experiments were pooled, in summary at least 183 cells of each genotype were analyzed for statistical differences by ANOVA followed by Bonferroni correction (SPSS 17.0, SPSS Inc.).

### Number and morphology of mitochondria

Mitochondria were stained with Mito Tracker Red (CM-H2XRos, 25 mM, Molecular Probes®, invitrogen™) and Mito Tracker Green FM (50 mM, Molecular Probes®, invitrogen™) in living motor neurons after 4 days in culture for 10 min After replacement of the medium, mitochondria were visualized with a fluorescence microscope (Axio Observer Z1, AxioCam MRm, Carl Zeiss MicroImaging GmbH) with incubation chamber for stable atmosphere (37°C, 5 % CO_2_). First, the number of mitochondria in a 10-μm distance of the proximal and distal axon was counted, and results pooled from four independent experiments. Altogether, at least 53 mitochondria in 18 different cells were counted for each genotype. Second, the length of mitochondria from seven independent experiments (at least 109 mitochondria for each genotype) was measured with Neurolucida 8 software (MBF Bioscience). The results are indicated as mean and standard deviation (SD). Statistical analysis by ANOVA and Bonferroni correction was carried out via SPSS 17.0 (SPSS Inc.).

### Time-lapse imaging

Mitochondria were stained and visualized as described above. Time-lapse images were acquired at a frequency of 1/15 Hz and exposure time of 355 ms. For statistical analysis of mitochondrial movements ten independent experiments were performed. For each genotype, more than 25 mitochondria were tracked both in anterograde and retrograde direction, and maximum velocity and average velocity of moving mitochondria were measured with freeware ImageJ software and the MTrackJ plug-in (http://rsbweb.nih.gov/ij/index.html). Statistical difference of mean and standard deviation was calculated by ANOVA and Bonferroni correction (SPSS 17.0, SPSS Inc.).

## Results

### Histological analysis of motor cortex, spinal cord, and muscle in *KIF5A*^*–/–*^ mice

Mice lacking KIF5A have a severe phenotype with death shortly after birth [29]. We analyzed cortex, spinal cord, and muscle paraffin sections of *KIF5A* ^*–/–*^ embryos (E 18.5) and confirmed normal morphology of cortex sections. Additionally, muscle tissue appeared normal in all three genotypes (exemplary images are shown in Online Resource 2). Previous qualitative histological analysis of brain and spinal cord in *KIF5A* ^*–/–*^ embryos suggested swelling of lower motor neuron cell bodies and nuclei, but quantitative data were missing [29]. In contrast to these data we found that the nuclear area was significantly smaller in *KIF5A* ^*–/–*^ lower motor neurons of the spinal cord in comparison to *KIF5A* ^*+/+*^ controls (115 ± 22 vs. 141 ± 27 μm^2^). Quantitative analysis of cell body area did not reveal significant differences between the genotypes, although there was a trend towards smaller cell bodies (241 ± 73 vs. 276 ± 82 μm^2^) in *KIF5A* ^*–/–*^ motor neurons.

### Reduced survival of *KIF5A*^*–/–*^ motor neurons

**Fig. 1:**
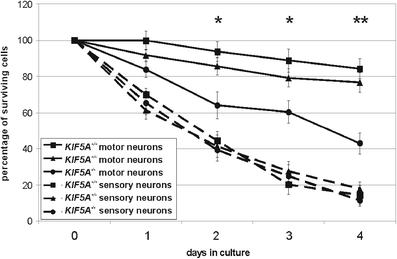
Survival of primary motor and sensory neurons from *KIF5A* ^*+/+*^, *KIF5A* ^*+/–*^, and *KIF5A* ^*–/–*^ mice. The number of initially counted cells was set 100 % (day 0). The percentage of surviving cells was calculated for every day in culture. Motor neurons are indicated in continuous lines, sensory neurons in broken lines. *Squares* show *KIF5A* ^*+/+*^, *triangles KIF5A* ^*+/–*^, and *circles KIF5A* ^*–/–*^ mice. After 4 days in culture mean survival of *KIF5A* ^*–/–*^ motor neurons had declined to 43 ± 6 %, whereas in *KIF5A* ^*+/–*^ 76 ± 5 %, and in *KIF5A* ^*+/+*^ mice 84 ± 6 % of the cells survived. In sensory neurons, there was no significant difference between the three genotypes at any time point. In total, at least 11 mice were analyzed per genotype. Mean and SEM are given. * denotes significant difference with *p* < 0.05 between *KIF5A* ^*+/+*^ and *KIF5A* ^*–/–*^ motor neurons, ** significant difference with p < 0.05 between *KIF5A* ^*+/+*^ and *KIF5A* ^*–/–*^, and *KIF5A* ^*+/–*^ and *KIF5A* ^*–/–*^ motor neurons

Motor and sensory neuron cell cultures were obtained from the lumbar spinal cord and the adjacent DRGs, dissected from day E 12.5 mouse embryos. The cells were grown on coverslips over 4 days in presence of neurotrophic factors. Cells were identified as motor and sensory neurons by morphology and positive anti-islet, anti-AChT, anti-MAP2ab, and anti-phospho-tau staining. Typical examples are shown in Online Resource 3. *KIF5A* ^*–/–*^ motor neurons showed reduced survival rates in comparison to heterozygous and wildtype littermates. After 4 days in vitro (DIV), mean survival of *KIF5A* ^*–/–*^ motor neurons had declined to 43 %, whereas in *KIF5A* ^*+/–*^ 77 %, and in *KIF5A* ^*+/+*^ mice 84 % of the cells survived (Fig. 1, continuous lines). In sensory neurons, there was no significant difference between the three genotypes (Fig. 1, broken lines).

### Reduced outgrowth of *KIF5A*^*–/–*^ motor and sensory neurons

**Fig. 2:**
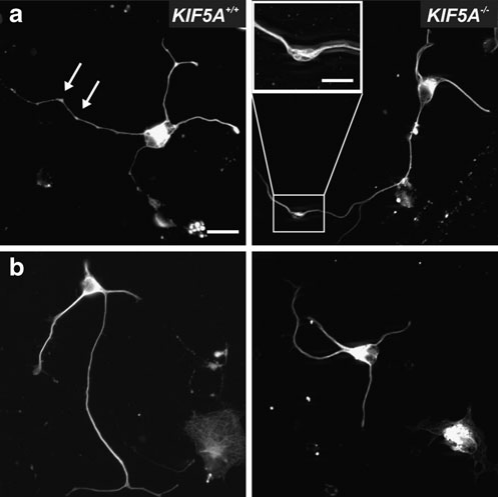
Morphology of *KIF5A* ^*+/+*^ and *KIF5A* ^*–/–*^ motor neurons. Motor neurons were stained with an antibody against neurofilament L (*white*). **a** Shows examples with axonal swellings (arrows, magnification box), **b** without axonal swellings. *Bar* 20 μm. *Bar in the magnification box* 10 μm

Fluorescence microscopy of *KIF5A* ^*–/–*^ motor neurons stained with antibodies against phospho-tau and MAP2ab revealed no gross morphological abnormalities (data not shown). Local accumulations of the cytoskeletal protein neurofilament L in axonal swellings showed a trend towards being more frequent in of *KIF5A* ^*–/–*^ motor neurons (12.4 ± 5.1 % of cells), in comparison to *KIF5A* ^*+/–*^ (9.3 ± 1.0 % of cells), and *KIF5A* ^*+/+*^ (4.3 ± 4.0 % of cells) motor neurons, but the difference did not reach statistical significance (Fig. 2). In sensory neurons, no gross morphological abnormalities were observed (data not shown).

**Fig. 3:**
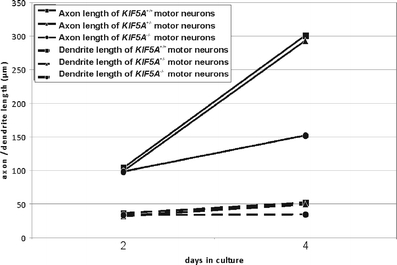
Axon and dendrite outgrowth of *KIF5A* ^*+/+*^, *KIF5A* ^*+/–*^, and *KIF5A* ^*–/–*^ motor neurons. Total axon length including all branches (*continuous lines*) and total dendrite length (*broken lines*) were measured after 2 and 4 days in vitro. *Squares* show *KIF5A* ^*+/+*^, *triangles KIF5A* ^*+/–*^, and *circles KIF5A* ^*–/–*^ motor neurons. Results are given as mean; due to clarity standard deviation indicators have been omitted. Measured values were linearly interpolated. For each genotype more than 150 cells from three independent experiments were analyzed

**Fig. 4:**
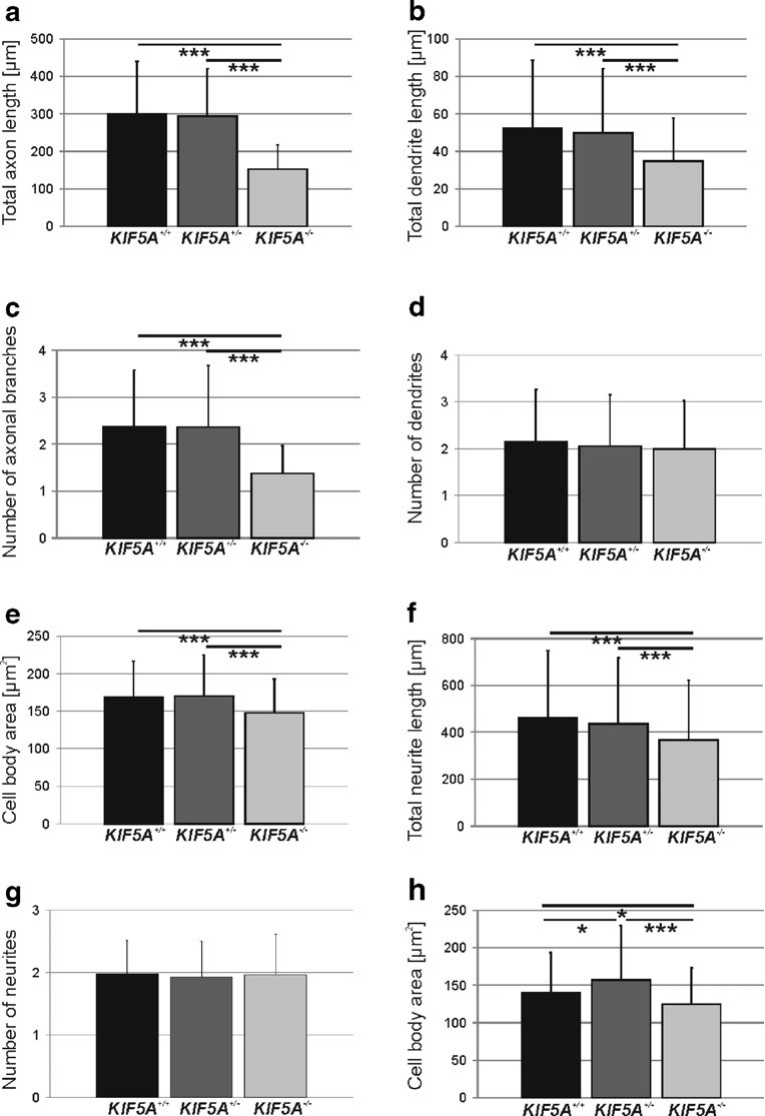
Process outgrowth of motor and sensory neurons from *KIF5A* ^*+/+*^, *KIF5A* ^*+/–*^, and *KIF5A* ^*–/–*^ mice. For each genotype more than 150 cells from three independent experiments were analyzed. Results are given as mean and standard deviation. Total axon length including all branches (**a**), total dendrite length (**b**), number of axonal branches (**c**), number of dendrites (**d**), and cell body area (**e**) are given for *KIF5A* ^*+/+*^, *KIF5A* ^*+/–*^, and *KIF5A* ^*–/–*^ motor neurons. Neurite outgrowth (**f**), number of neurites (**g**), and cell body area (**h**) in *KIF5A* ^*+/+*^, *KIF5A* ^*+/–*^, and *KIF5A* ^*–/–*^ sensory neurons. *p < 0.05, ***p < 0.001

Since in HSP, the motor neurons with the longest axons are preferentially affected, we assessed the influence of *KIF5A knockout* on process outgrowth. Motor neurons were fixed after 2 and 4 days in culture respectively, and axons were stained with an anti-phospho-tau antibody. Dendrites were stained by an anti-MAP2ab antibody. After 2 days in vitro, there was no significant difference neither in axonal nor in dendritic outgrowth. At that time mean axonal length was approximately 100 μm, total dendrite length about 35 μm (Fig. 3). After 4 days in culture, axonal and dendritic outgrowth was impaired in *KIF5A* ^*–/–*^ motor neurons. Total axon length (including all branches) in motor neurons was significantly reduced in *KIF5A* ^*–/–*^ vs. *KIF5A* ^*+/–*^, and *KIF5A* ^*+/+*^ motor neurons (Fig. 4a). Similar results were obtained for the longest axonal branch (data not shown). As in axons, the total dendrite length (per cell) was significantly lower in *KIF5A* ^*–/–*^ motor neurons vs. heterozygous and wildtype neurons (Fig. 4b). Also, the longest dendrite was reduced in *KIF5A* ^*–/–*^ motor neurons in comparison to controls (data not shown). There was a significant reduction in the number of axonal branches in *KIF5A* ^*–/–*^ vs. *KIF5A* ^*+/–*^ and *KIF5A* ^*+/+*^ motor neurons (Fig. 4c), whereas the number of dendrites was not altered (Fig. 4d). Cell body area was significantly reduced in *KIF5A* ^*–/–*^ motor neurons (Fig. 4e).

Sensory neurons were fixed after 24 h in culture as neurites in sensory neurons develop more rapidly than in motor neurons. In sensory neurons, the total neurite length was significantly diminished in *KIF5A* ^*–/–*^ cells (Fig. 4f). Similarly, the longest neurite was significantly shortened in *KIF5A* ^*–/–*^ sensory neurons compared to controls (data not shown). The number of neurites was not altered between the genotypes (Fig. 4g). In *KIF5A* ^*–/–*^ sensory neurons cell bodies were smaller than in *KIF5A* ^*+/+*^ and *KIF5A* ^*+/–*^ sensory neurons. Surprisingly, cell bodies of *KIF5A* ^*+/–*^ sensory neurons were larger than in wildtype (Fig. 4h).

### Axonal transport in motor neurons

As axonal transport disturbances of mitochondria may lead to a reduced number of organelles in a length-dependent manner, we counted mitochondria in proximal and distal parts of the axon. The overall number of mitochondria in a 10 μm segment of the proximal and distal end of an axon did not differ between the genotypes and ranged about seven to ten mitochondria in the proximal part and six to seven mitochondria in the distal part. Detailed results are shown in Table 1. Since damaged mitochondria often are fragmented and present with a more round appearance whereas viable mitochondria show an elongated shape [32, 33] we assessed mitochondrial morphology as a potential indicator of impaired mitochondrial function. In axons of motor neurons the mean length of mitochondria did not significantly differ between *KIF5A* ^*–/–*^ (1.3 ± 0.7 μm), *KIF5A* ^*+/–*^ (1.2 ± 0.7 μm) and *KIF5A* ^*+/+*^ mice (1.4 ± 0.8 μm).

**Table 1:** Number of mitochondria in a 10 μm segment of the proximal and distal end of an axon in *KIF5A* ^*+/+*^, *KIF5A* ^*+/–*^, and *KIF5A* ^*–/–*^ motor neurons

Number of mitochondria	*KIF5A* ^*+/+*^	*KIF5A* ^*+/–*^	*KIF5A* ^*–/–*^
Proximal axon (10 μm)	10.2 ± 4.7	7.9 ± 4.6	7.4 ± 2.8
Distal axon (10 μm)	6.0 ± 2.4	6.6 ± 2.3	6.0 ± 3.8

Mean and standard deviation are given. Mitochondria were counted in at least 18 different cells for each genotype

**Fig. 5:**
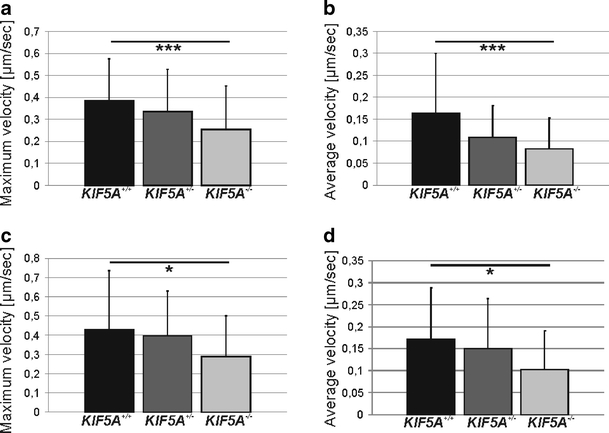
Axonal transport of mitochondria in motor neurons. Maximum and average velocity of mitochondrial transport in anterograde (**a** and **b**) and retrograde (**c** and **d**) direction in *KIF5A* ^*+/+*^, *KIF5A* ^*+/–*^, and *KIF5A* ^*–/–*^ motor neurons. Mean and standard deviation are given. *p < 0.02, ***denotes *p* < 0.001. Results from ten independent experiments were summarized

To assess mitochondrial transport as a critical factor of energy supply especially in distal parts of the axon and at the synapse, we analyzed mitochondrial movements in axons of motor neurons both in anterograde and retrograde direction using time-lapse imaging. Tracking of mitochondrial movements over 30 minutes revealed a transport deficit in *KIF5A* ^*–/–*^ motor neurons. The maximum velocity and the average velocity of (moving) mitochondria in anterograde direction were significantly impaired in *KIF5A* ^*–/–*^compared to *KIF5A* ^*+/+*^ motor neurons. *KIF5A* ^*+/–*^ showed an intermediate velocity of mitochondria in motor neurons (Fig. 5a,b). The absence of KIF5A led also to a reduction in retrograde transport velocity, with the maximum velocity of retrogradely transported mitochondria being significantly reduced in comparison to *KIF5A* ^*+/+*^ cells. Similarly, the average velocity decreased in *KIF5A* ^*–/–*^ cells in comparison to *KIF5A* ^*+/+*^ cells (Fig. 5c,d, see also Online Resources 4, 5, and 6 for movies of mitochondrial movements in motor neurons).

## Discussion

Dysfunctional KIF5A causes spastic paraplegia (SPG10) with predominant motor neuron affection in humans. To improve the understanding of the underlying pathophysiology, we characterized the effect of loss of KIF5A on survival, morphology, and axonal transport in motor neurons in a *KIF5A* ^*–/–*^ mouse model. In summary, we observed impaired axonal transport, reduced axonal outgrowth, and reduced survival of primary motor neurons lacking KIF5A.

### KIF5A knockout leads to an axonopathy, rather than a neuronopathy

Although these observations fit to a primary axonal affection the possibility that early axonal changes are just an expression of general dysfunction of motor neurons in *KIF5A* ^*–/–*^ mice cannot be completely ruled out. However, several observations argue for axonopathy as the primary problem caused by the lack of KIF5A.

- Axon and dendrite growth is not generally impaired but becomes obvious only when processes exceed a critical length. After 2 days in culture processes have a similar length in all genotypes. Especially axons and (to a lesser extent) dendrites in *KIF5A* ^*+/+*^ and *KIF5A* ^*+/–*^ motor neurons elongate considerably within the next two days, whereas process length barely increases in *KIF5A* ^*–/–*^ motor neurons (Fig. 3).
- The observation of reduced survival of *KIF5A* ^*–/–*^ motor neurons in culture would fit to both a primary axonal defect and general motor neuron dysfunction. However, the observation of a normal motor cortex and at most minor changes in spinal motor neurons in late embryonic *KIF5A* ^*–/–*^ mice despite a perinatally lethal dysfunction of motor neurons (probably due to impaired innervation of respiratory muscles) are in agreement with an axonopathy.
- Furthermore, the observation of an increased number of axonal swellings in cultured motor neurons lacking KIF5A points to primary axonal impairment and is in line with autopsy findings of spastic paraplegia, e.g., in SPG4, the most frequent form of autosomal dominant HSP in which impaired microtubule severing is thought to cause primary axonal disturbance [34]. For SPG10, no pathoanatomical studies are available.
- Defects of anterograde and retrograde transport as observed in our study but also in squid and drosophila models lacking kinesin-1 are in line with primary axonopathy. Anterograde transport deficit is likely to explain limitations in axonal outgrowth whereas the additional deficit of retrograde transport on the long run may cause impaired growth factor supply to the neuron that may contribute to reduced survival of *KIF5A* ^*–/–*^ motor neurons.

### KIF5A/KHC disruption impairs axonal transport both in anterograde and retrograde direction

To assess axonal transport as a potentially critical factor in the extreme anatomy of motor neurons we tracked mitochondrial movements in motor neurons with time-lapse microscopy. Interestingly, we found a significant reduction of transport velocity in both directions, anterograde and retrograde.

Perturbance of anterograde transport seems to be plausible for a motor protein involved in fast anterograde transport, whereas disturbance of retrograde transport is surprising at first glance. In accordance with our findings, involvement of kinesin-1 both in anterograde and retrograde transport has been shown in three additional models: Perfusion of squid axoplasms with an antibody against KHC inhibited rate and number of anterogradely and retrogradely moving organelles [35, 36]. *Drosophila* flies lacking the KIF5A homologue KHC also have an axonal transport deficit for mitochondria both in the anterograde and retrograde direction [37, 38]. In the *KIF5A KO* mouse model, neurofilament transport frequency and velocity is reduced in both directions [30]. The human N256S mutation of the *KIF5A* gene in a mouse model causes a reduced frequency of neurofilament transport in both directions, whereas transport velocity is increased in retrograde and normal in anterograde direction [31].

### Anterograde and retrograde transport are interdependent

Different scenarios could explain the axonal transport impairment in both directions. First, if mitochondria are produced in or near the cell body, they have to be transported properly to the periphery before they can head back to the cell body. This fact alone seems not sufficient to explain our results since the number of mitochondria in the periphery is not altered. Second, the retrograde motor protein dynein is synthesized in the cell body and needs to be transported to distal parts of the axon before participating in retrograde transport. This hypothesis is supported by the dependence of anterograde dynein transport on kinesin-1 [36, 39]. Third, KIF5A could act as a biochemical or biophysical activator of dynein in axons. In the recent years, the concept of cargoes concomitantly bound to motors for both directions has been accepted [40]. The exact mode of motor coordination and regulation of axonal transport direction and velocity is still unresolved. Experimental data examining whether the number of active motors influences transport velocity and length are controversial [41–43]. In vitro data suggested a role of the neck/linker region of KIF5A proteins for transport velocity regulation [44], but this hypothesis was not supported by in vitro experiments utilizing a human SPG10 mutation in the neck domain of the KIF5A protein [28]. The number of other factors implicated in kinesin activity and transport dynamics is increasing steadily and depends on the cargo [45]. The interdependence of KIF5A and dynein is also enforced by the fact that disruption of dynein function by using RNA interference inhibited neurofilament transport both in anterograde and retrograde direction [30]. Mitochondrial transport seems to be influenced amongst other factors by mitochondrial membrane potential [46], nerve growth factor [47], dynamin-related protein-1 [48], and phosphatidylinositol [49]. But a global plan of the interactions has not yet emerged. The concept of “communicating motors” would be able to explain the observed results in squid axoplasm, *Drosophila KHC KO* and *KIF5A KO* mouse model.

Beyond mitochondria for power supply and neurofilaments as part of the cytoskeleton, the members of the kinesin-1-family transport a variety of other cargoes, also including tubulin [24], synaptic vesicles [22, 23], and lysosomes [21]. These cargoes could also be critical for neuronal survival, outgrowth, and synaptic function. However, a detailed understanding of the full repertoire of cargoes transported by KIF5A is not yet available and requires further work.

### Lack of KIF5A affects motor neurons more severely than sensory neurons

The outgrowth of processes is impaired in motor neurons of *KIF5A* ^*–/–*^ mice but affects axons more severely than dendrites. In addition, axon length has to exceed a critical length until the outgrowth defect gets obvious. This nicely parallels the situation in patients with SPG10 where the longest axons of the corticospinal tract innervating the legs are mostly affected, whereas the shorter axons to the arms are mainly spared. If the length of processes is critical, the involvement of other cell types with long processes has to be expected as well. Indeed, in sensory neurons which have axons of similar length as motor neurons we found reduced outgrowth as well, even though to a lesser extent than in motor neurons. This would also be concordant to the human disease: patients with SPG10 may have additional sensory deficits, but much less severe than motor affection. The cause of primary involvement of motor neurons is not known but may be due to special functions executed by specific linking or binding proteins, higher need of common cell functions, e.g., increased metabolic demand, and/or a lower reserve capacity. However, a high variability in the age at onset and disease severity even within one family has been reported for the HSPs, including SPG10, so additional factors seem to influence the development of the disease.

### Pathogenesis of SPG10

Whether KIF5A mutations cause SPG10 by a lack of functional kinesin (haploinsuffiency) or by a dominant negative effect is not quite clear. The majority of mutations in SPG10 patients are missense mutations in the motor domain of kinesin which is responsible for binding of kinesin to microtubules and its movement along microtubules. Indeed, functional in silico analyses found that kinesin with different missense mutations causing SPG10 in humans leads to a reduced binding to microtubules or reduced transport velocity, respectively [28]. The observation that *KIF5A* ^*+/–*^ mice with reduced amounts of KIF5A protein developed normally also argues against haploinsufficiency as the major mutational effect, but favors a dominant negative effect. Additionally, we found a severe deficit in survival, outgrowth, and mitochondrial transport in *KIF5A* ^*–/–*^ motor neurons, whereas *KIF5A* ^*+/–*^ motor neurons did not considerably differ from wildtype cells, so half the normal amount of KIF5A seems to be sufficient to maintain neuronal functions. In vivo, the kinesin-1 complex is a heterotetramer made up of two kinesin heavy chains and two light chains. In the SPG10 situation, dimers could include two functional wildtype KIF5A proteins, one wildtype and one mutated KIF5A or two mutated non-functional KIF5A proteins. In case of a dominant negative effect the motor neuron would be left with only 25 % of normal kinesin. Whether this drastic decrease of functional kinesin is causing disease or, alternatively, microtubules become blocked from dysfunctional kinesin causing a traffic jam or, as a third scenario, abnormal kinesin binds to cargo and blocks its processing, remains to be established.

Our animal model hints at an involvement of mitochondrial transport in the pathogenesis of some forms of HSPs. This might be a link to other HSP forms that are caused by mitochondrial dysfunction, e.g., SPG7 and SPG13 with mutations in a mitochondrial protease and chaperone, respectively.

In summary, our results point out a role of KIF5A in process outgrowth and axonal transport of mitochondria, affecting motor neurons more severely than sensory neurons. This gives pathophysiological insights into KIF5A associated HSP, and matches the clinical findings of predominant degeneration of the longest axons of the corticospinal tract.

## Electronic supplementary material

Below is the link to the electronic supplementary material.

Supplementary Fig. 1 Measurement of neuronal processes. **a** Immunocytochemical staining of a motor neuron with antibodies against MAP2ab (*red*) and phospho-tau (*green*). *Bar* 20 μm. The process length was analyzed as follows: **b** longest axonal branch, **c** total axon length including all its branches, **d** longest dendrite, **e** cell body area (JPEG 23 kb)

High-resolution image (TIFF 1075 kb)

Supplementary Fig. 2 Immunohistochemistry of *KIF5A* ^*+/+*^ and *KIF5A* ^*-/-*^ mice. **a** Cortex, **b** muscle, and **c** spinal cord paraffin sections were stained with cresyl violet (**a**, **c**) and hematoxylin eosin (**b**) respectively. No gross morphological changes were found but nuclear area was smaller in *KIF5A* ^*–/–*^ lower motor neurons of the spinal cord in morphometric analysis. *Bar* 20 μm (JPEG 61 kb)

High-resolution image (TIFF 1168 kb)

Supplementary Fig. 3 Immunocytochemistry of *wildtype* motor and sensory neurons. Examples for motor neurons are shown in the left panel, for sensory neurons in the right panel. **a**–**c** anti-MAP2ab-staining in red. **a** anti-islet, **b** anti-AChT, **c** anti-phospho-tau staining in green. *Bar* 20 μm. (JPEG 36 kb)

High-resolution image (TIFF 1433 kb)

ESM 4 (MPG 2072 kb)

ESM 5 (MPG 2074 kb)

ESM 6 (MPG 2074 kb)
