# Supplementary figures and images for: Axonal transport deficit in a KIF5A–/– mouse model
Source: Neurogenetics. 2012 Apr 1;13(2):169–79. doi: 10.1007/s10048-012-0324-y (PMC3332386; doi:10.1007/s10048-012-0324-y)

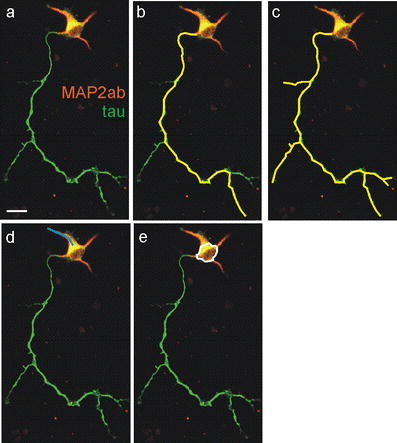

Supplement: Supplementary file 1 — Measurement of neuronal processes. a Immunocytochemical staining of a motor neuron with antibodies against MAP2ab (red) and phospho-tau (green). Bar 20 μm. The process length was analyzed as follows: b longest axonal branch, c total axon length including all its branches, d longest dendrite, e cell body area (JPEG 23 kb) [file 10048_2012_324_Fig6_ESM.jpg]

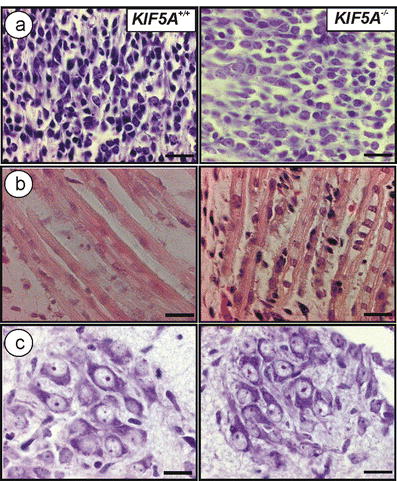

Supplement: Supplementary file 3 — Immunohistochemistry of KIF5A +/+ and KIF5A -/- mice. a Cortex, b muscle, and c spinal cord paraffin sections were stained with cresyl violet (a, c) and hematoxylin eosin (b) respectively. No gross morphological changes were found but nuclear area was smaller in KIF5A –/– lower motor neurons of the spinal cord in morphometric analysis. Bar 20 μm (JPEG 61 kb) [file 10048_2012_324_Fig7_ESM.jpg]

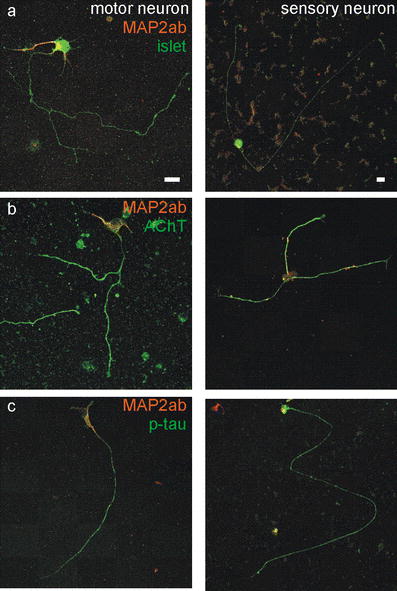

Supplement: Supplementary file 5 — Immunocytochemistry of wildtype motor and sensory neurons. Examples for motor neurons are shown in the left panel, for sensory neurons in the right panel. a–c anti-MAP2ab-staining in red. a anti-islet, b anti-AChT, c anti-phospho-tau staining in green. Bar 20 μm. (JPEG 36 kb) [file 10048_2012_324_Fig8_ESM.jpg]
